# Medical Philosophy

**Published:** 1891-08

**Authors:** L. B. Anderson

**Affiliations:** Norfolk, Va.


### MEDICAL PHILOSOPHY.

BY L. B. ANDERSON, M. D., NORFOLK, VA.

All true philosophy is based on facts. The object of every philosopher, therefore, is to ascertain facts, and by logical deductions therefrom, to reach fundamental principles.

Every fact is indestructable, and therefore eternal, and must of necessity harmonize with all other facts. Were all the well authenticated facts which are strewn along the pathway of medicine collected, arranged and co-ordinated, there would be a canon to which every devotee of medical science could appeal in testing the validity of every new hypothesis. In the absence of such an arbiter, medical history is ever repeating itself, and the so-called scientists of to-day are constantly announcing discoveries as new and reliable, which were published, accepted or rejected, ages ago. Despite the want of such a canon, there are great fundamental facts accessible to every physician, which logical deductions and accurate observation may enable him to utilize, and render him an independent, self-poised, reliable and successful practitioner.

"Principles, not men," was the adage of an old political philosopher, which is as essential in medicine as in politics. Of what avail is an appeal to so-called medical "authorities," when their opinions rest alone on their *ipse dixit*, instead of clearly defined principles, logically deduced from well-established facts? To illustrate:

Just nine years ago it was announced that Koch had discovered the pathogenic cause of pulmonary consumption, in the "bacillus tuberculosis." This being assumed as a fact (though its absurdity was so apparent that it was almost self-evident), the general deduction therefrom was, that any agent which would destroy the bacillus would cure the consumption. And the medical world eagerly sought for the desired agent. It was just the other day the startling announcement was made, that "Koch had at last discovered a cure for consumption." and the civilized world rang with the praises of the great bacteriologist. But at the late meeting of the International Medical Congress, Koch himself made the astounding revelation that his "remedy acted upon the diseased tissue, and not on the bacilli." In this declaration Koch surrenders the whole question of bacterial pathogenesis. For if "the bacilli are not acted on, by the remedy," and their presence does no harm, then they cannot be pathogenic. And if they die after the removal of the diseased tissue, it is plain, as the writer has always contended, that they are a *creatnre*, and not a *cause*, of the disease. And yet, with this open confession of failure, both as to pathology, etiology and therapeutics, how humiliating is the attitude of the great medical profession of the world to-day! Even the learned editor of Gaillard's Medical Journal, a graduate of our great University, himself a Virginian, with all the conservatism for which our people are famous, seems to burst into ecstacy at the very mention of the name of Koch. "We owe," he says, "a debt of gratitude to the great mind which has conceived, and the bold hand which has executed the magnificent experiment in the interest of suffering humanity. We, as a profession, give Koch reverence for the courage, unselfishness and devotion to science which he has manifested. Amid all the glamour of fame, the acclamation of nations, the blessings of sufferers, the curses of envious men, he stands calm and unruffled, and continues his labors of love, unmoved by sordid ambition or petty revenge. We, too, may say with Gossler, medicine is 'fortunate to call such a son its own.'"

One, in reading this grand apostrophe, would suppose that all of Koch's declarations were built upon facts, and his conclusions were logical deductions from the same, and that he is indeed a medical philosopher. But hear him again: "It (the lymph) is a dangerous remedy in many cases—thus far not one positive case of cure has been reported. We read of 'apparent cures;' of 'improvement,' all cautiously worded. The tocsin of triumph has not yet been sounded. May we not listen in vain for its glad tidings!" This is the measure of the success of one who sits "calm and unruffled amid the glamour of fame, the acclamation of nations and the blessings of sufferers"—not one of whom has been cured. If medicine rests upon no surer foundation or sounder philosophy than this, it must ever remain an imposition and a cheat. So much for the philosophy of the learned editor referred to. But what of the great mass of the profession and press of the world? Always professing to oppose quackery, charlatanry and secret nostrums, and reading out of the profession any member who would endorse them, and yet to day they are bowing in adoration at the shrine, measured by the standard of medical ethics, of the greatest, but Hahnemann, the world has ever seen. He deals out his secret nostrum by the drop, and everybody seems eager to procure and use it, without knowing any more of its composition or modus operandi than he does of Jayne's expectorant or Morris' panacea. Yet the one is extolled to the skies, while the others are damned to Tophat. Oh! consistency, thou art a jewel!

In order to demonstrate how unphilosophical are the principles and practices of the great body of our medical savants, we will make a brief review of bacteriology, or that branch of science which, in its various phases, absorbs the professional mind more generally than any others. There are certain assumptions in relation to bacteria, which are now conceded by nearly all scientists to be well established facts, viz.:

1. That bacteria are microscopical vegetations.

2. That they are governed by the same laws which govern all the higher orders of vegetation.

3. All plants and trees have their respective habits, due to climate, soil, moisture, without which they wither and die.

4. No plant can perform its life-processes in a soil deficient in the elements essential to its growth.

5. A micro-coccus is the microscopical seed of any bacillus while in a dormant state.

6. Every seed has a nucleus or kernel composed of the embryo in albumen, enveloped in a capsule.

7. On the application of heat, moisture, and the contact of hydrogen, with the exclusion of light, all seeds will begin the process of germination, which ceases as soon as the contained albumen is consumed, unless it be in soil affording the nutriment necessary to its growth.

With these facts before us, let us examine the micrococcus, or germ of the bacillus tuberculosis.

The first important fact connected with the life-history of the *micrococcus bacilli tuberculosis* is, that it has never been known to develop into a bacillus, except in association with tuberculous matter. Hence, if the cause of tuberculosis, then it has to germinate the soil for its own life process, which would be in direct conflict with the laws of all vegetable growth. Then, such an assumption would be a self-evident absurdity. If, on the other hand, they could germinate a soil for their own growth, and such soil be the veritable pulmonary tubercle, it is evident that not one animal that breathes, from man to a mouse, could escape tuberculosis. The correctness of these conclusions are verified by many additional facts, viz.: Bacilli may be sought in vain in an acid urine; but if it be rendered alkaline, and placed in certain surroundings favorable for fermentation or putrefaction, it will soon swarm with bacteria, demonstrating that the soil produces the bacteria, and not the bacteria the soil. If a certain variety of bacteria exists in a culture of a given character, and the character of the culture be changed by the addition of other elements, the existing bacteria will either be supplanted by a new and different colony, or they will be so changed in their characteristics as to be unrecognizable. Specimens of laudable pus, freshly drawn from a gonorrhœal urethra, or an abscess, will present no bacteria under the field of the microscope, but let it remain sufficiently long for chemical laws to establish putrefaction, and thousands of bacteria will appear. This I have verified so often that I can affirm it with the most positive assurance' As with Pasteur, Koch, Cohn, Cheyne, and every bacteriologist of any note, I have never found bacteria in healthy fluids· blood or tissue, so I have never seen them, or even any indications of their presence, except where fermentation, putrefaction or regeneration exists.

To say bacteria or their germs do produce putrefaction in animal, or fermentation in vegetable substances, is as unphilosophical and illogical as to attribute to them pathogenic influences. We know that all organic matter, so soon as the vital laws which affect and perpetuate its existence cease to operate, becomes at once subject to chemical laws, under the operation of which new and less complex bodies are formed, with the evolution of mephitic gases and putrescent fluids, ptomoids and leucomaines, when, and not before, micrococci, not finding a soil congenial to their germination, rapidly develop into bacteria. So soon as these putrid emanations, on which bacteria feed, are consumed, the bacteria die. The only ostensible mission of bacteria, therefore, is to consume dead and decomposing matters, whether in or out of the body; hence, instead of being esteemed *hastes humani generis*, they should be hailed as heaven-sent scavengers to rid our bodies and the world of death-dealing ptomaines and leucomaines. Well has Trouessant said: "The part played by microbes in nature is an important one. They are nourished at the expense of organic matters in a state of putrefaction; in this way they clear the earth from dead bodies and fœcal matter; from all dead and useless substances which are the refuse of life."

To illustrate the pure hypothetical, and hence unphilosophical and illogical basis on which bacterial pathogenesis rests, we quote from Koch's report as published in the *American Journal of the Medical Sciences*, 1884, pp. 188–189: "It is needless to say that in extensive examinations of healthy but non-tubercular tissue these bacilli (tubercular) were never met with. The bacilli of tuberculosis are limited to a parasitic mode of life in animals and cannot grow outside of the body under the conditions found in nature" But on the next page, to sustain his theory of bacterial pathogenesis, he says: "The bacillary invasion of healthy tissues invariably precedes the tubercular invasion." Here the great authority in bacteriology, Koch, himself, makes two statements of FACTS, which are diametrically opposed to each other, antagonizing the fundamental principles of philosophy, viz.: that all facts are indestructible, eternal, and hence harmonious and therefore synergetic. Mark the phraseology of the great dreamer: "I have never found bacilli in healthy or non-tubercular tissue." Then: "The bacillary invasion of healthy tissue invariably precedes the tubercular invasion." How is it possible for him to affirm "that bacilli invade healthy tissues before tubercles are developed," when he declares that "he has never found them in healthy tissues?" To say that he means that bacilli invade tissues affected with other diseases than tuberculous, and therein produce tubercle, makes it no better, for he as distinctly affirms that "he has never met with them in *morbid tissues* other than tubercular."

The sum of the whole matter, when tested by the principles of logic, is this: If bacterial germs cannot germinate in healthy fluids and tissues, it is demonstrable that they cannot produce disease therein, for it is impossible for an agent to perform any function where it cannot live. And if they can only live in diseased or decaying fluids and tissues, and die as soon as they wander therefrom, or the food therein is consumed, it is evident they are the offspring and not the cause of such disease or degeneration. And since they only germinate in and consume the products of disease, decay and putrefaction, it is self-evident that, instead of being pathogenic, their functions are salutary and sanative.

In apparent conflict with all these facts and deductions, we are confronted with the assumed fact that a pure culture of bacillus anthrax, diphtheria, tuberculosis, or cholera, will, when injected into a healthy animal, produce a similar disease in it. Therefore, it is concluded that bacilli are pathogenic.

Bear in mind that bacilli can only live in septic matter, and hence they can be injected into living tissues only in the matter in which they live. And if we can prove that such matter, deprived of microbes, bacillary or bacterial life, when injected into a healthy animal, will produce therein a disease identical with that from which it sprang, we will have abolished the last and most specious subterfuge of the advocates of bacterial pathogenesis. Then to the facts and the philosophy.

Dr. Richard Ward Richardson, of London, than whom I know of no profounder medical philosopher, conducted a series of experiments, extending through a period of seven years, and after years of close observation, he finds the conclusions then drawn have been the more confirmed and established in his convictions. He transmitted from animal to animal, by the inoculation of various morbid secretions, diseases of a specific character. In order to eliminate all possible suspicion of microbic influence, he reduced the secretions to a crystalline alkaloid l salt, and in every instance an inection of a solution of this alkaloid in water produced the specific disease, while an inoculation with the secretions of the animal thus affected produced the same disease in other animals. The poison from the eye of a person suffering from contagious ophthalmia passes to the eye of another person; presently there is a free secretion, which, in turn, becomes affected and poisonous. It is not that the particle of poison has propagated a new particle, but it is that the natural secretion of the eyeball has come in contact with an atom of poisonous matter, and immediately at the point where the atom of poison was implanted, there is a change in the secretion. The process widens the circle, more secretion pours out, more poison is produced, and the increase goes on until at last the whole body of the animal may become affected by absorption of poisonous matter in the blood from the injured surface."

Just a few days ago Gartenlaube, of Leipzig, in writing on diphtheria in animals, said: "The poison of (the) diphtheria (bacillus) has been isolated by Briegen as a whitish albuminous mass, soluble in water, and termed toxalbumen. Hypodermic injections of this poison in animals produces symptoms of diphtheria at intervals, proportioned to the strength of the case, although it is perfectly free from bacilli."

Since the septic matter of diphtheria, puerperal peritonitis, etc., "perfectly free from bacilli," or reduced to an alkaloid state, in the form of pure crystals, and injected into a healthy animal, will produce sepsis in that animal, while the secretions from such an animal will produce a similar trouble in another animal when inoculated with them, therefore it is the specific matter, and not the bacteria, which is the pathological agent.

Having viewed this question in a philosophical and logical manner from every conceivable standpoint, and reached the same conclusion from all, we are drawn to the inevitable conclusion that bacterial pathogenesis has no foundation in logic, philosophy or fact.

In order to test the validity of this final conclusion by its practical application in the most important operations known to surgery, we will view its results in the hands of two of the greatest laparotomists the world has ever seen—one standing at the head, the other at the foot of the line.

Without precedents, anesthetics or antiseptics, with few and rude instruments, and inexperienced assistants and nurses, a son of Virginia, Ephraim McDowell, himself not a graduate in medicine, in the backwoods of Kentucky, more than eighty years ago, performed thirteen laparotomies, several of them of the most formidable character, with nine perfect recoveries.

Lawson Tait, who, measured by his vast experience and great success, stands head and shoulders above any living surgeon, while the lesser stars which shine so dimly around him in the medical firmament as scarcely to be seen, are bathing their hands and seething their ligatures and instruments in torrid germicidal waters, pursues the even tenor of his way, using in his major operations only the ordinary drinking water from the tap for cleansing, and any cotton batting for dressing. As to bacterial pathogenesis, he says: "My fear of germs has steadily diminished, so that if I could get them in sufficently large quantities, and found them dry and elastic, I would willingly stuff my pads with them instead of wool." A few months ago Dr. Tait wrote to me thus: "I use none of the usual germicidal washes, nothing but plain water from a tap. The advances in so-called 'bacteriology' have had nothing to do with surgical success. My success would have been as great, and I think more rapid, if Lister and his devices had never been heard of. Listerism stood in the way from 1877 to 1882, as a bugbear." From Dr. Reginald Harrison, formerly of Liverpool, but now of London, I received a communication expressing similar views, while Dr. Bantock, of London, as my friend, Dr. Lankford informs me, entertains the views and adopts the methods of Dr. Tait.

One word as to Koch's lymph. It is not a germicide. It contains no bacteria. It is evidently an organic alkaloid of most potentially poisonous qualities. It acts upon the human system whether sick or well, whether tuberculous or not tuberculous. It is claimed to act specifically on tuberculous tissue. These are admitted facts. It is, however, said to act specifically upon lupus, and lupus is claimed to be tuberculous. With this declaration I take issue, but will not pause to discuss it. It is said that it excites a high degree of inflammation in the diseased tissue, excites healthy action in the contiguous healthy tissue, cuts off supplies from the diseased tissue and causes it to slough off and become a healthy, granulating surface which restores the part to health.

Admitting this to be so, it is no more than is often accomplished by purely local means—arsenious acid, chloride of lime, beach drop and powdered blood-root, for instance—in lupus, and by various constitutional means in pulmonary tuberculosis. But after all, it is a total surrender of Koch's previous claims of the necessity and efficacy of germicides in curing diseases claimed to be the result of bacterial pathogenesis.

